# ADH1B Arg47His Polymorphism Is Associated with Esophageal Cancer Risk in High-Incidence Asian Population: Evidence from a Meta-Analysis

**DOI:** 10.1371/journal.pone.0013679

**Published:** 2010-10-27

**Authors:** Guohong Zhang, Ruiqin Mai, Bo Huang

**Affiliations:** 1 Department of Pathology, Shantou University Medical College, Shantou, China; 2 Department of Laboratory Medicine, the First Affiliated Hospital, Shantou University Medical College, Shantou, China; University of Medicine and Dentistry of New Jersey, United States of America

## Abstract

**Background and Objectives:**

Incidence of Esophageal squamous cell carcinoma (ESCC) is prevalent in Asian populations, especially in the ones from the “Asian esophageal cancer belt” along the Silk Road and the ones from East Asia (including Japan). Silk Road and Eastern Asia population genetics are relevant to the ancient population migration from central China. The *Arg47His* (rs1229984) polymorphism of *ADH1B* is the highest in East Asians, and ancient migrations along the Silk Road were thought to be contributive to a frequent ADH1B*47His allele in Central Asians. This polymorphism was identified as responsible for susceptibility in the first large-scale genome-wide association study of ESCC and that's explained by its modulation of alcohol oxidization capability. To investigate the association of *ADH1B Arg47His* with ESCC in Asian populations under a common ancestry scenario of the susceptibility loci, we combined all available studies into a meta-analysis.

**Methods:**

A dataset composed of 4,220 cases and 8,946 controls from twelve studies of Asian populations was analyzed for *ADH1B Arg47His* association with ESCC and its interactions with alcohol drinking and *ALDH2 Glu504Lys*. Heterogeneity among studies and their publication bias were also tested.

**Results:**

The ADH1B*47Arg allele was found to be associated to increased risk of ESCC, with the odds ratios (OR) being 1.62 (95% CI: 1.49–1.76) and 3.86 (2.96–5.03) for the *His/Arg* and the *Arg/Arg* genotypes, respectively. When compared with the His/His genotype of non-drinkers, the *Arg/Arg* genotype can interact with alcohol drinking and greatly increase the risk of ESCC (OR = 20.69, 95%CI: 5.09–84.13). Statistical tests also showed gene-gene interaction of *ADH1B Arg+* with *ALDH2 Lys+* can bring more risk to ESCC (OR  = 13.46, 95% CI: 2.32–78.07).

**Conclusion:**

Revealed by this meta-analysis, ADH1B*47Arg as a common ancestral allele can significantly increase the risk of ESCC in Asians, especially when coupled with alcohol drinking or the ALDH2*504Lys allele.

## Introduction

Esophageal carcinoma (EC) is ranked as the eighth most common malignancy and the seventh leading cause of cancer death worldwide, and among EC the esophageal squamous cell carcinoma (ESCC) is a predominant histological type. The epidemiology of ESCC is characterized by remarkable differentiation in incidence against geographical distribution and ethnic backgrounds. It is known that Asian countries, especially China, Iran, and Japan, have the highest rates of ESCC over the world. Such highest incidences are noted as the famous “Asian esophageal cancer belt” that ranges from the Caucasian mountains, across northern Iran, and all through the way to northern China [1]. This “Asian esophageal cancer belt” extends along the ancient Silk Road, which was established by the Han Dynasty of China for contact with Central Asia about 2000 years ago. Studies have shown that there exists extensive genetic admixture in populations along the Silk Road region [2]. Another high-incidence area for ESCC is Japan and Korea of East Asia, where populations have been probed to be genetically close to the ancient populations in north-central China [3]. In China, the Taihang Mountain region of north-central China has the highest incidence of ESCC. Ancestors of the Chaoshan Han Chinese migrated from that region to their current habitat about 2000 years ago [4], and now Chaoshan is shown to be another high-incidence area for ESCC. Historical records about 2,000 years ago have noted several unique epidemiological features of EC, and it was described in the text of Yi-Guan as “commonly seen in elders and rarely developing in young people” and referred to as “Ye Ge”, which means dysphagia and belching.

The metabolism of alcohol has two steps and involves alcohol dehydrogenase-1B (*ADH1B*) and aldehyde dehydrogenase-2 (*ALDH2*), two genes of enzymatic oxidation. Upon consumption of alcoholic beverage, ethanol is first catalytically oxidized into acetaldehyde, mainly through *ADH1B*. Acetaldehyde is subsequently metabolized into harmless acetate, chiefly by *ALDH2*. A polymorphism at codon 47 in exon 3 of the *ADH1B* gene, resulting in an amino acid transition from arginine (*Arg*) to histidine (*His*), brings super-active metabolization of ethanol. About a 40-times maximum velocity has been identified for the fast *His/His* genotype of *ADH1B* compared to the less active *Arg/Arg* form [5], [6]. Global distribution of the frequency for ADH1B*47His allele shows a clear east-to-west decline, where specifically it is dominant in East/West Asia, moderate in southeast Asia and rare in other continents [7], [8], [9], [10]. Such a distribution pattern was considered to be related to the selection of the *Arg47His* polymorphism of *ADH1B*, which was along the emergence and expansion of rice domestication in East Asia [11].

As for *ALDH2*, a polymorphism resulting from the substitution of glutamate (*Glu*) by lysine (*Lys*) at residue 504 (also recognized as *Glu487Lys*) makes the new sequence encode a catalytically inactive subunit, whose respective *ALDH2 Glu/Lys* genotype has only 6.25% effectiveness of the normal *ALDH2 Glu* protein [12]. The ALDH2*504Lys allele is essentially absent all over the world except in East Asia and has its highest frequency in China [13]. That is why the alcohol flushing response is called Asian Glow, where the ALDH2*504Lys allele serves as a biomarker [14] The existence of this mutant allele in East Asia was considered the result of Han Chinese' population and migration in the region [13].

Various studies focused on *ADH1B* polymorphisms and its relationship to the risk of ESCC in Asian high-incidence populations [15], [16], [17], [18], [19], [20], [21], [22], [23], [24], [25], [26]. A significantly increased risk was found for ADH1B*47Arg when combined with moderate or heavy alcohol consumption. For example, *ADH1B Arg/Arg* was found associated with 1.2 and 74 times increased risk in non-drinkers and drinkers, respectively, compared to non-drinkers with the *ADH1B His/His* genotype [19]. A meta-analysis of 7 studies on Asians (Chinese, Japanese, and Thailand) revealed the ALDH2*504Lys allele can bring more risk to ESCC susceptibility [27]. Some of these studies also assessed the effect of gene-gene interaction between the *ADH1B Arg47His* and *ALDH2 Glu504Lys* polymorphisms on ESCC risk [22].

Sample size issues are always hard to completely overcome due to reasons like budgets and sampling chances, which often make conclusions of some studies not replicable in the others. In search of a better precision under a wider scenario, we here performed a meta-analysis based on surveyed eligible studies to examine the association between *ADH1B Arg47His* polymorphism and ESCC risk in high-incidence Asian populations.

## Materials and Methods

### Selection of eligible studies

Candidate studies were identified by searching the PubMed and MEDLINE for relevant articles in English. The latest searches were performed on Jun 21, 2010. The following terms were jointly used for searching: “oesophageal cancer” or “esophageal cancer” and “*ADH2*” or “aldehyde dehydrogenase” or “*ADH1B*” or “polymorphism”. Search results were filtered to include only studies involving subjects of Asian populations. The eligible for our meta-analysis were then all population-based case-control studies reporting association between *ADH1B Arg47His* variants and ESCC. Review articles, case-only articles, esophageal adenocarcinoma case-control and repeated literatures were excluded. A search of cited reference of retrieved articles was also carried out to reduce the possible bias of coverage due to searching only the two databases. A total of 12 studies examining the association of the *ADH1B Arg47His* polymorphism were found, out of which 3 articles analyzed the interaction between *ADH1B* and *ALDH2* genes.

### Data extraction

The following information was extracted from each eligible study: author, year of publication, country of origin, characteristics of cancer cases and controls, and genotypic information.

### Meta-analysis

A χ2 test was performed to examine Hardy-Weinberg equilibrium when genotypic data were available. The risks of ESCC added by the *ADH1B Arg47His* variant were estimated for each study and the metadata as well via odds ratio (OR) using the Comprehensive Meta-analysis software version 2.0. Specifically, the risks of the variant genotypes (*Arg/Arg+Arg/His*) in contrast to that of the His/His genotype were firstly evaluated. Then the ORs of *Arg/Arg* versus *His/His* were calculated. Between-study heterogeneity was estimated using Cochran's Q, a χ2-type statistic. Heterogeneity was considered statistically significant when P<0.05. Point estimates and 95% confidence intervals (CI) were computed under both the random effect and the fixed effect models. If heterogeneity existed, point estimates and 95% CIs were estimated using the random effect model, otherwise the fixed effect model was employed. Sensitivity analysis was performed by omitting an individual study each time to find potential outliers to the pooled OR. For the analysis of gene-gene interaction between *ADH1B Arg47His* and ALDH2*504Lys, the *His/His* and *Glu/Glu* was considered the reference genotype. For gene-environment interaction, individuals of the *His/His* genotype with non-drinker label were set as reference subjects. For each category of alcohol drinking, there is no common standard for classification, so we only selected the heavy alcohol drinking as the analysis category. Publication bias was assessed using funnel plots and Eggers test.

## Results

### Eligible studies and meta-analysis databases

Table 1 tabulated the characteristics of the all studies included in this meta-analysis. Twelve studies published in 1997 through to 2010 were about the relationship between the *ADH1B Arg47His* polymorphism and ESCC risks. From these studies, a dataset with 4,220 cases and 8,946 controls (Table 1) was established. Six studies (1,332 cases/2,384 controls) were conducted in China. Another five (2,145 cases/5,191 controls) were performed in Japan. The last study concerning ESCC was from Iran, which has 743 cases and 1,371 controls. The genotype distribution in the control group in each study did not depart from HWE with P<0.05 (data not shown).

**Table 1:** Information of studies included in meta-analyses of ADH1B Arg47His polymorphism on ESCC risk.

First author [Ref]	Year	Country (Province)	cases	controls	Crude OR	
					Arg/Arg vs.His/His	Arg/His vs.His/His
**Ding** [**15**]	2009	China(Jiangsu)	191	221	2.42(1.02−5.77)	1.30(0.85−1.99)
**Oze** [**16**]	2009	Japan(Aichi)	585	1170	3.25(2.22−4.76)	1.32(1.07−1.63)
**Cui** [**24**]	2009	Japan(unknown)	1067	2763	4.10(3.24−5.18)	1.17(1.00−1.37)
**Akbari** [**17**]	2009	Iran(Golestan)	743	1371	2.06(1.25−3.39)	1.71(1.03−2.85)
**Guo** [**18**]	2008	China(Gansu)	80	480	1.46(0.71−2.59)	3.67(1.26−8.73)
**Lee** [**19**]	2008	China(Taiwan)	406	656	6.09(4.10−9.03)	1.30(0.98−1.72)
**Yang** [**20**]	2007	China(Sichuan)	191	198	1.91(0.92−3.95)	1.89(1.10−3.22)
**Chen** [**21**]	2006	China(Taiwan)	330	592	5.65(3.67−8.69)	1.22(0.90−1.65)
**Wu** [**22**]	2005	China(Taiwan)	134	237	6.89(3.52−13.49)	1.52(0.94−2.47)
**Yang** [**25**]	2005	Japan(Aichi)	165	494	1.12(0.44−2.86)	2.07(1.44−2.99)
**Yokoyama** [**26**]	2002	Japan(unknown)	234	634	5.728(3.49−9.39)	1.16(0.82−1.62)
**Hori** [**23**]	1997	Japan(Tokyo)	94	130	6.15(2.41−15.67)	1.65(0.91−2.99)

Unknown: no special province.

### Heterogeneity result

To determine the amount of heterogeneity that existed among the 12 studies, we did a Cochran's Q test. No heterogeneity was found (p = 0.46; Q = 10.81 on 11 df) in the analysis for *Arg/Arg*+*Arg/His* vs. *His/His* genotype, so we chose the fixed-effect model over the random-effect model. However, test results indicated that analyses for *Arg/Arg* vs. *His/His* genotype in different studies were heterogeneous (p<0.05; Q = 34.62 on 11 df). Hence, for this part of comparison the random-effect model was selected.

### Publication bias

Funnel plot and Egger's test were performed to assess the publication bias. The shape of the funnel plot (Figure S1) did not indicate any obvious evidence of asymmetry. Additionally, no significant publication bias was found in the results of Egger's test: p is 0.09 for *Arg/Arg*+*Arg/His* vs. *His/His* and 0.52 for *Arg/Arg* vs. *His/His*, respectively.

### Meta-analysis results

Shown in Figures S2 and S3 are the core results of this meta-analysis. When the homozygotes (*Arg/Arg*) and all Arg carriers (*Arg/Arg* +*Arg/His*) were compared with the homozygotes of His/His, significant differences were found between the groups of cases and controls. The pooled ORs for all the 12 studies were 1.62 (95% CI: 1.49–1.76, p<0.001) for the fixed model and 3.86 (95% CI: 2.96–5.03, p < 0.001) for the random model, respectively.

### Sensitivity analyses

Influence of each study on the pooled OR was examined by repeating the meta- analysis with one study excluded at each time. Results (data not shown) show that there is no significant change of the pooled OR, and thus indicates the robustness of our findings.

### Gene-environment interaction

In Table 2, the impact of combined genotypes on ESCC risk stratified by alcohol drinking is presented as ORs and 95% CI. Compared with non-drinkers who carried the *ADH1B His/His* genotype, drinkers with the *ADH1B Arg/Arg* or the *Arg/His* genotype can experience increased ESCC risk by 20.69 and 6.97 fold, respectively. Combined result of *Arg/His*+*Arg/Arg* vs. *His/His* suggests that there is a significant association of the genotype with ESCC risk in drinkers (OR: 7.34, 95% CI: 2.10–25.71).

**Table 2:** Interaction between alcohol drinking and ADH1B genotypes on ESCC.

Genotype (ref[15], [19], [21])	Non-drinker			Drinker		
	Case/control	OR	95% CI	Case/control	OR	95% CI
His/His	101/515	ref	-	270/241	4.94	1.16−20.96
Arg/His	84/415	1.06	0.75–1.5	278/176	6.97	1.70–28.56
Arg/Arg	16/51	2.08	1.10–3.92	208/42	20.69	5.09–84.13
Arg/His+ Arg/Arg*	143/529	1.17	0.88–1.55	556/253	7.34	2.10–25.71

*: genotype data from the reference 15, 19, 20, 21.

The next step was to obtain more accurate estimates for the combined risk of *ADH1B Arg/Arg* genotype and alcohol drinking. It was reported that the drinking status was related to the risk of ESCC, which was also confirmed in our above results. Since the standards for categorizing alcohol drinking status varied in literatures [18], [19], [21], [22], we selected the category of heavy drinkers with the *ADH1B Arg/Arg* genotype from each of the literature as the case group, and set non-drinkers with the *ADH1B* His/His genotype as the reference group. Such a comparison reveals heavy-drinkers with the *ADH1B Arg/Arg* genotype had a 70.12-fold increase (95% CI: 40.60–121.10) of risk for ESCC (Figure S4).

### Gene-gene interaction

Table 3 shows the result of interaction analysis of *ADH1B* and *ALDH2* on ESCC risk. Compared to the subjects having *ADH1B His/His* and *ALDH2 Glu/Glu*, ORs for those with *ALDH2 Glu/Glu* and *ADH1B* Arg+, *ALDH2* Lys+ and *ADH1B His/His*, and *ALDH2 Lys+* and *ADH1B Arg+* were 3.66–13.46. The most significantly increased risk for ESCC risk (OR: 13.46; 95% CI: 2.32–78.07, p<0.001) was noted in individuals with *ADH1B Arg+* and *ALDH2 Lys+*.

**Table 3:** Gene-gene interaction of ADH1B and ALDH2 on ESCC risk.

Genotype[15], [19], [22]	Case	Control	P value	OR	95% CI
**Glu/Glu and His/His**	134	299	ref	-	-
**Glu/Glu and Arg+**	301	196	0.0001	3.66	1.66–6.74
**Lys+ and His/His**	82	69	0.005	2.72	1.34–5.51
**Lys+ and Arg+**	181	50	0.004	13.46	2.32–78.07

## Discussion

### A Common ancestralness and homogeneity of ESCC susceptibility locus in Asian population

Some alleles that increase the risk of complex diseases like cancers, are ancestral [28]. Both historical records of migration and genetic fingerprints of East Asians and ancient population in Central China suggested there were some common ancestries in populations along the “Asian esophageal cancer belt”. The ADH1B*47His allele is highly prevalent in Asian populations, particularly in northeast Asians (i.e., Chinese, Japanese, and Koreans). It was proposed the ADH1B*47His allele may serve better as an ancestral marker for understanding expansions and migrations of ancient Central China populations, and is related to disease susceptibility and natural selection as well [29]. Except for in East Asia, a high frequency of the ADH1B*47His allele is also found in West Asian countries such as Iran and Turkey, where high-incidence regions of ESCC exist, too [9]. Such a worldwide double-peak pattern of frequency distribution of the ADH1B*47His allele was argued to be the result of independent increase of the derived allele in both western and eastern Asia after humans had spread across Eurasia [9]. Based on these facts and findings, we propose that ADH1B*47Arg is a risk factor for ESCC in Asian populations that stems from recent common ancestralness of them.

Above meta-analysis was conducted under such a scenario and the results gave quite solid conclusions over the association of ADH1B*47Arg with increased ESCC risks. The pooled ORs for response were 1.62 (95% CI: 1.49–1.76, P< 0.001) and 3.86 (95% CI: 2.96–5.03, p < 0.001) for *Arg/Arg* homozygotes and Arg carriers, respectively. Analysis also showed no heterogeneity was found among all the twelve studies (Q = 9.59 on 10 df; p = 0.48), suggesting there can be more collabrations in studies of high-incidence Asians on the way to find other common ancestral loci that is responsible for the etiology of ESCC.

### Gene-environment interaction with alcohol drinking

Many studies have noticed that the synergistic interaction between *ADH1B* polymorphism and alcohol drinking may exist in increased risk of ESCC[18], [21], [22]. In this meta-analysis, the ESCC risk of individuals with the *ADH1B Arg/His* genotype and alcohol drinking is increased by 6 fold. Thus, reduced *ADH1B* enzyme activity and resulted higher concentration of alcohol can be a very important risk factor for ESCC. Abstinence from alcohol might be the most effective solution for ADH1B*47Arg carriers to reduce their risks of developing into ESCC.

### Gene-gene interaction of ADH1B Arg47His with ALDH2 Glu504Lys

The two genes, *ADH1B* and *ALDH2,* have previously been identified for susceptibility in the first genome-wide association study of ESCC [24]. Many studies explored the interaction between the *ADH1B Arg/His* and the ALDH2*504Lys polymorphisms [18], [19], [22]. In this meta-analysis, higher ESCC risk groups are *ADH1B Arg+* and *ALDH2 Lys+* (OR  = 13.46, 95% CI: 2.32–78.07), which indicates a strong interaction between them. Therefore carriers having both *ADH1B Arg+* and *ALDH2 Lys+* bear much higher risk of ESCC.

### Limitations

Although our primary result of this meta-analysis is constructive, some limitations still exist. First, we lacked the genotype data from populations in central Asia such as Kazakhestan, Uzbekistan and Turkamanistan and the data of Chaoshan Chinese in China. Second, the criteria for categorizing drinking levels are different across studies. And the last, OR value was obtained without correction over age, gender or other environmental factors. Adjustments over these covariates might help better disect the association between *ADH1B Arg47His* and the susceptibility of ESCC.

### Conclusion

Our study demonstrated significant association between the ADH1B*47Arg allele and the increased risk of ESCC in high-incidence Asian populations. Together with alcohol drinking, especially heavy alcohol drinking, the *Arg/Arg* genotype can greatly increase the ESCC risk. This effect can be further strengthened by the gene–gene interaction between the *ADH1B Arg47His* and the *ALDH2 Glu504Lys* polymorphisms. A meta-analysis of association under a common ancestralness perspective did provide valuable clues in clarifying the etiology puzzle of ESCC.

## Supporting Information

Figure S1 Funnel plot of publication bias in all studies by Comprehensive Meta-analysis software. Log OR of ESCC is plotted versus standard error for each of the 12 studies in this meta-analysis. Each point represents a separate study for the indicated association by Arg/Arg+Arg/His over His/His genotype.
(1.31 MB TIF)

Figure S2 Forest plot of the odds ratios and confidence intervals of the association of Arg/His+Arg/Arg over His/His genotype.
(0.98 MB TIF)

Figure S3 Forest plot of the odds ratios and confidence intervals of the association of Arg/Arg over His/His genotype.
(1.33 MB TIF)

Figure S4 Forest plot of the odds ratios and confidence intervals of the interaction between Arg/Arg genotype and heavy alcohol drinking.
(1.04 MB TIF)
