# Supplementary figures and images for: ADH1B Arg47His Polymorphism Is Associated with Esophageal Cancer Risk in High-Incidence Asian Population: Evidence from a Meta-Analysis
Source: PLoS One. 2010 Oct 27;5(10):e13679. doi: 10.1371/journal.pone.0013679 (PMC2965113; doi:10.1371/journal.pone.0013679)

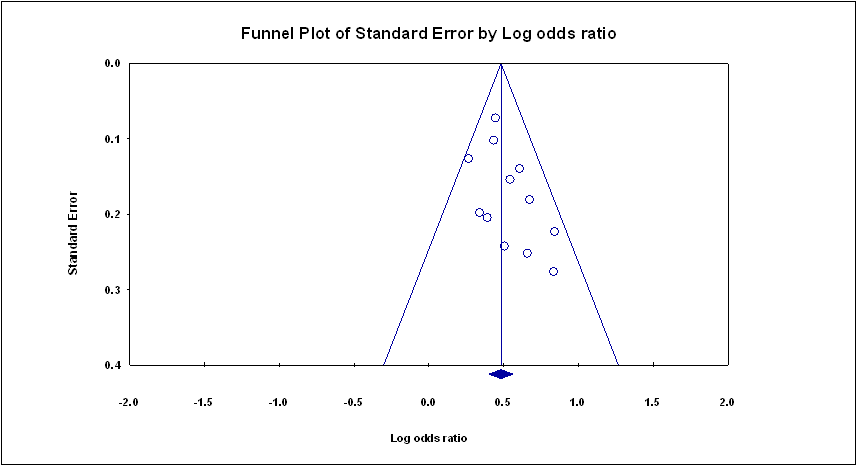

Supplement: Figure S1 — Funnel plot of publication bias in all studies by Comprehensive Meta-analysis software. Log OR of ESCC is plotted versus standard error for each of the 12 studies in this meta-analysis. Each point represents a separate study for the indicated association by Arg/Arg+Arg/His over His/His genotype. (1.31 MB TIF) [file pone.0013679.s001.tif]

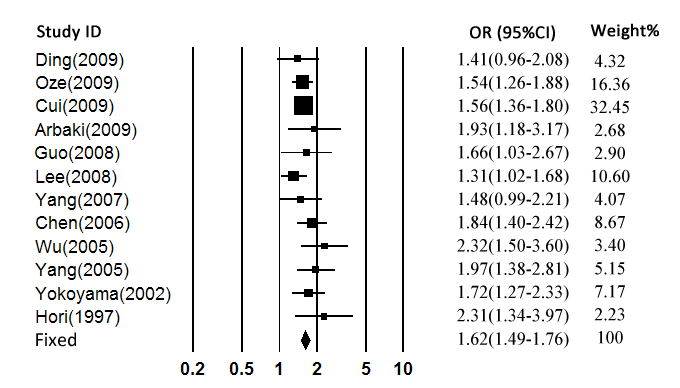

Supplement: Figure S2 — Forest plot of the odds ratios and confidence intervals of the association of Arg/His+Arg/Arg over His/His genotype. (0.98 MB TIF) [file pone.0013679.s002.tif]

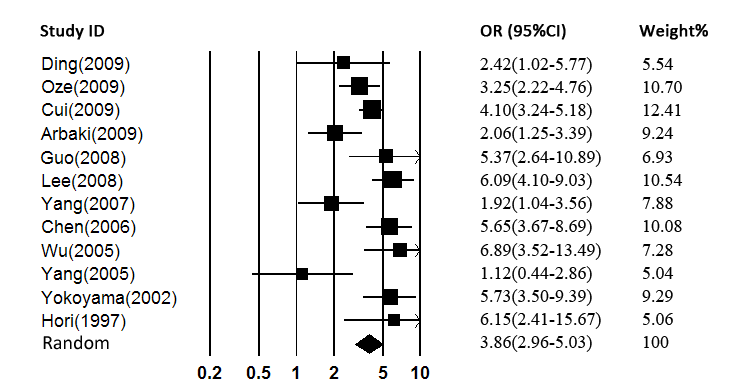

Supplement: Figure S3 — Forest plot of the odds ratios and confidence intervals of the association of Arg/Arg over His/His genotype. (1.33 MB TIF) [file pone.0013679.s003.tif]

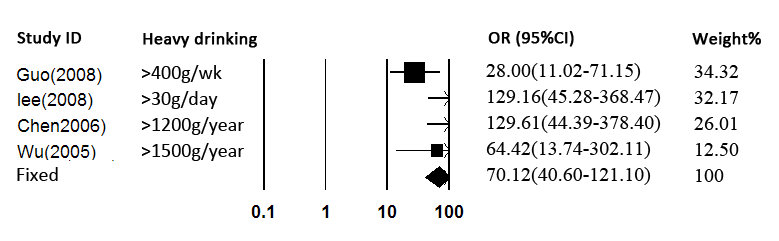

Supplement: Figure S4 — Forest plot of the odds ratios and confidence intervals of the interaction between Arg/Arg genotype and heavy alcohol drinking. (1.04 MB TIF) [file pone.0013679.s004.tif]
